# Muscle weakness as an additional criterion for grading sarcopenia‐related prognosis in patients with cancer

**DOI:** 10.1002/cam4.4362

**Published:** 2021-12-10

**Authors:** Emanuele Cereda, Richard Tancredi, Catherine Klersy, Federica Lobascio, Silvia Crotti, Sara Masi, Silvia Cappello, Nicole Stobäus, Maja Tank, Sara Cutti, Luca Arcaini, Elisabetta Bonzano, Sara Colombo, Paolo Pedrazzoli, Kristina Norman, Riccardo Caccialanza

**Affiliations:** ^1^ Clinical Nutrition and Dietetics Unit Fondazione IRCCS Policlinico San Matteo Pavia Italy; ^2^ Medical Oncology Unit Fondazione IRCCS Policlinico San Matteo and Department of Internal Medicine University of Pavia Pavia Italy; ^3^ Biometry and Clinical Epidemiology Service Fondazione IRCCS Policlinico San Matteo Pavia Italy; ^4^ Clinical Research Unit Charité – Universitätsmedizin Berlin, corporate member of Freie Universität Berlin Humboldt‐Universität zu Berlin and Berlin Institute of Health Berlin Germany; ^5^ Medizinisches Versorgungszentrum Hämatologie‐Onkologie Tempelhof Berlin Germany; ^6^ Research Group on Geriatrics Charité – Universitätsmedizin Berlin, corporate member of Freie Universität Berlin Humboldt‐Universität zu Berlin and Berlin Institute of Health Berlin Germany; ^7^ Medical Direction Fondazione IRCCS Policlinico San Matteo Pavia Italy; ^8^ Division of Hematology Fondazione IRCCS Policlinico San Matteo and Department of Molecular Medicine University of Pavia Pavia Italy; ^9^ Radiation Oncology Department Fondazione IRCCS Policlinico San Matteo and University of Pavia Pavia Italy; ^10^ Department of Nutrition and Gerontology German Institute of Human Nutrition Potsdam‐Rehbrücke Nuthetal Deutschland

**Keywords:** bioelectric impedance analysis (BIA), cancer, handgrip strength, mortality, prognosis, sarcopenia

## Abstract

**Background:**

Low muscle strength has been pointed out as a key characteristic of sarcopenia, but the prognostic significance of muscle function next to reduced skeletal muscle mass (SMM) in patients with cancer has been scantily investigated.

**Methods:**

Data on muscle strength by handgrip (HG) dynamometry and total‐body SMM estimated by bioelectrical impedance analysis (BIA) of Italian and German patients with cancer observed prospectively until death or censoring were analysed (*N* = 1076). Patients were stratified in four risk categories based on low HG (<10th percentiles of age and gender‐specific normative values) and low total‐body SMM according to SMM index cutoffs (<10.75 and <6.75 kg/m^2^ in men and women, respectively).

**Results:**

During a median follow‐up of 58 months [25th–75th percentile, 37–60], 566 patients had died. Patients presenting low HG in combination or not with low SMM were characterised by shorter median survival (12.7 vs. 27.2 months, respectively; *p* < 0.001) compared to those with low SMM/normal HG and normal SMM/normal HG (>60 months for both). After adjusting for sex, age, body mass index and percentage of weight loss, disease's stage, performance status and type of cancer, compared to reference category (normal HG and SMM; *N* = 210) the hazard ratios were: low SMM/normal HG (*N* = 342), 0.83 [95% confidence interval, CI, 0.67–1.02] (*p* = 0.073); normal SMM/low HG (*N* = 158), 1.19 [95% CI, 1.07–1.32] (*p* = 0.002); low SMM/low HG (*N* = 366), 1.39 [95% CI, 1.27–1.53] (*p* < 0.001).

**Conclusions:**

Muscle weakness was found to be a more powerful predictor of survival than BIA‐estimated SMM and should be considered as an additional key feature of sarcopenia in patients with cancer.

## Abstract 2

Low muscle strength has been pointed out as a key characteristic of sarcopenia, but the prognostic significance of muscle function next to reduced skeletal muscle mass (SMM) in patients with cancer has been scantily investigated. Muscle weakness was found to be a more powerful predictor of survival than SMM and should be considered as additional key feature of sarcopenia in patients with cancer.

## 1 INTRODUCTION

In the last two decades, there has been growing interest in the prognostic impact of body composition in patients with cancer. Changes in body composition are heterogeneously associated with unintentional weight loss and of multifactorial origin, but mainly caused by a combination of reduced food intake and metabolic processes related not only to the tumor inflammatory burden, but also to anticancer treatments.^1^, ^2^, ^3^ Specifically, reduced skeletal muscle mass (SMM)––namely sarcopenia––has been associated with increased mortality and chemotherapy‐related toxicity independently of body mass index,^4^, ^5^, ^6^, ^7^ which is a relevant issue, given the current overweight/obesity epidemic.^8^ However, although sarcopenia has received an independent International Classification of Disease‐10 code as a condition characterised by low muscle mass and weakness,^9^ literature shows some inconsistencies in its definition. In patients with cancer, the diagnosis of sarcopenia has been always based on the presence of low muscle mass, while in old adults its identification requires the presence of low muscle strength in combination with muscle changes, with poor physical performance as indicative of a severe condition.^10^, ^11^, ^12^ Interestingly, recent studies have addressed and highlighted that also reduced muscle function could have a negative and independent prognostic impact, resulting in increased mortality, dose‐limiting toxicity and other perioperative outcomes (e.g. length of stay, readmission rate),^13^, ^14^, ^15^, ^16^, ^17^ particularly when reduced strength and lean/muscle mass coexist.^13^, ^14^, ^15^ Its use as alternative phenotypic criterion of malnutrition for indicating reduced muscle mass––rather than other body composition parameters, such as arm muscle circumference, fat‐free mass index and muscle mass by computed tomography (CT)––has been also tested, showing a stronger prediction of mortality in patients with cancer.^18^, ^19^, ^20^ As muscle strength testing could be an informative, low‐cost, well accepted and routinely feasible procedure,^21^ further study in this area are warranted.

In this cohort study, we investigated the prognostic role of handgrip strength in addition to body composition in patients with cancer.

## 2 SUBJECTS AND METHODS

### 2.1 Participants

Study participants were Italian and German patients with cancer, consecutively included in previous intervention trials (NCT02055833; NCT02065726; NCT02828150)^22^, ^23^, ^24^ and cohort studies^25^, ^26^, ^27^ and prospectively followed at the Fondazione IRCCS Policlinico San Matteo (Pavia, Italy) and the Charité – Universitätsmedizin Berlin (Berlin, Germany). In these studies, common inclusion criteria were: age ≥18 years, solid or haematological neoplastic disease, complete data on most relevant clinical features (anthropometry, cancer type, stage and performance status), exposure (handgrip strength and body composition) and outcome (vital status). We excluded patients with implanted pacemakers or defibrillators, oedema or ascites as these factors can interfere with body composition assessment by bioelectric impedance.

### 2.2 Assessments

The following data were collected at first evaluation:

1. Demographic and major clinical information: cancer site, disease's stage (American Joint Committee on Cancer stage groupings) and performance status (Eastern Cooperative Oncology Group [ECOG]).^28^
2. Anthropometry: body weight (to the nearest 0.1 kg) and height (to the nearest 0.5 cm); in body mass index (BMI; calculated as weight [kg]/height [m]^2^); and percentage of unintentional weight loss (%WL) occurred in the previous 6 months.^29^ Then, patients were graded in 5 BMI‐adjusted WL risk categories using the 5 × 5 matrix proposed by Martin et al.^30^ and based on BMI (<20.0, 20.0–21.9, 22.0–24.9, 25.0–27.9 and ≥28.0 kg/m^2^) and %WL (≤−2.5%, −2.5% to −5.9%, −6.0% to −10.9%, −11.0% to −14.9% and ≥−15.0) strata.
3. Body composition: bioelectrical impedance analysis (BIA) was used to estimate total‐body SMM, using the equation of Janssen et al.^31^

$$\begin{array}{c} \text{SMM (kg)}=[\left({\text{height}}^{2}/\text{Resistance 5}0\;\text{kHz}\times 0.401\right)+\\ \left(\text{gender}\times 3.825\right)‐\left(\text{age}\times 0.071\right)]\;+5.102 \end{array}$$

[height, in sm; Resistance, in Ohms; gender, men = 1 and women = 0; age, in years]

We used the phase‐sensitive device NUTRILAB Akern srl in Italians (accuracy for resistance, 1%) and the multi‐frequency device Nutriguard–M Data Input GmbH in Germans (accuracy for resistance, ±0.5%). The two instruments have been derived from and cross‐validated against one another. Although a significant difference in the measure of 50 KHz‐resistance between the two devices (approximately +9 Ohms for the German technology) has been found,^32^ we have estimated that, using the equation of Janssen et al.,^31^ this would translate in a mean discrepancy in SMM < 0.5 kg. Furthermore, the diagnostic validity of BIA against CT for the assessment of muscle mass (MM) in patients with cancer has been recently reported.^33^ Its accuracy in identifying patients with low MM has been also demonstrated in the intensive care setting.^34^

Therefore, SMM index (SMMI) was calculated (SMM [kg]/height [m]^2^) and BIA‐derived low SMMI in men and women was defined by a value <10.75 and <6.75 kg/m^2^, respectively.^35^

- Muscle strength: handgrip strength (HG) was measured by hand dynamometry (DynEx™; Akern/MD Systems in Italians; Jamar, Sammons Preston Rolyan in Germans) in the dominant hand, testing the patient in sitting position with the shoulder adducted and neutrally rotated, the elbow flexed at 90°, and the forearm and wrist in neutral position. The mean of three consecutive trials was used in the analysis. Low HG was defined as a value <10th percentiles of age and gender‐specific normative values.^36^

### 2.3 Outcome ascertainment

Patients were actively followed (up to January 2020) until death or censoring (date of last contact) using the following methods: linkage to municipal registries, in‐office visits, inquiries by mail or telephone to participants or proxy respondents.

### 2.4 Statistical analysis

Descriptive statistics were provided for continuous (mean and standard deviation or median and interquartile range) and categorical variables (count and percentage). Patients were stratified in four categories by the presence of low SMMI and low HG. Differences between groups were investigated using the one‐way analysis of variance (continuous variables) and the Fisher's exact test (categorical variables). We used the reverse Kaplan–Meier method to calculate median follow‐up and survival curves were provided for SMMI/HG strata. Mortality rates (per 100 person‐year) and median survival, together with their 95% confidence interval (95% CI) were also computed. A multivariable model (Cox's regression) was used to evaluate the independent association of SMMI/HG strata and mortality. The following noncollinear confounders were included: sex, age, ECOG performance status, disease's stage, BMI‐adjusted weight loss risk categories and cancer site. Hazard ratio (HR) and 95% CI were computed. Huber–White's robust standard error was used to account for intra‐centre correlation. The interaction of SMMI and HG was assessed.

Data were analysed using the software STATA 16.1 (Stata Corporation) setting statistical significance to a two‐sided *p* level of <0.05.

## 3 RESULTS

In total, 1218 patients were enrolled in the study and 1076 were eligible for analyses (Italians, *N *= 450; Germans, *N* = 626). Reasons of exclusion were: lost to follow‐up, *n* = 1; missing data in handgrip strength, *n* = 141. Italian patients were mainly evaluated at diagnosis, while Germans were evaluated at different stages of the disease's course. The two cohorts presented heterogeneous and different cancer diagnoses but comparable stage. Italians were characterised by better performance status, although they presented lower muscle strength reasonably due to higher age, lower BMI and more frequent unintentional WL.^37^, ^38^

Reduced SMMI was found in 708 patients and 524 showed muscle weakness. The characteristics (clinical and nutritional) of the study population by low SMMI and HG strata are reported in Table 1. Reduced muscle mass and muscle weakness were associated with all demographic and clinical variables. Particularly, low HG was associated with WL, reduced performance status and advanced disease's stage. Furthermore, the risk of combined low muscle mass and strength was twice higher in male patients.

**TABLE 1:** Features of the study cohort by reduced skeletal muscle mass and muscle weakness

Demographic and clinical characteristic	Whole cohort (*N* = 1076)	Normal SMMI Normal HG (*N* = 210)	Low SMMI Normal HG (*N* = 342)	Normal SMMI Low HG (*N* = 158)	Low SMMI Low HG (*N* = 366)	*p*‐value^a^
Sex (male), *N* (%)	636 (59.1)	55 (26.2)	238 (31.8)	54 (34.2)	289 (79.0)	<0.001
Age (years), median (IQR)	64.7 (55.0–72.1)	64.2 (54.0–70.0)	66.0 (56.0–72.8)	61.3 (52.0–68.2)	65.0 (55.3–74.0)	0.23
Body mass index (kg/m^2^), mean (SD)	23.6 (4.3)	25.7 (4.4)	23.9 (4.1)	24.5 (4.9)	21.8 (3.5)	<0.001
6‐month weight loss (%), mean (SD)	8.6 (9.6)	5.8 (8.7)	6.9 (8.8)	9.2 (8.9)	11.4 (10.3)	<0.001
Cancer site, *N* (%)						<0.001
Gastrointestinal	343 (31.9)	72 (34.2)	133 (38.9)	39 (24.7)	99 (27.1)	
Head and neck	229 (21.3)	40 (19.1)	79 (23.1)	25 (15.8)	85 (23.2)	
Urogenital	157 (14.6)	35 (16.7)	35 (10.2)	37 (23.4)	50 (13.7)	
Haematological	144 (13.4)	35 (16.7)	47 (13.7)	22 (13.9)	40 (10.9)	
Neuroendocrine, adrenal and thyroid	37 (3.4)	10 (4.8)	5 (1.5)	10 (6.3)	12 (3.3)	
Lung	75 (7.0)	8 (3.8)	22 (6.4)	11 (7.0)	34 (9.3)	
others	91 (8.5)	10 (4.8)	21 (6.1)	14 (8.9)	46 (12.6)	
Cancer stage, *N* (%)						0.002
I	138 (12.8)	30 (14.3)	51 (14.9)	10 (6.3)	47 (12.8)	
II	107 (10.0)	29 (13.8)	42 (12.3)	8 (5.1)	28 (7.7)	
III	180 (16.7)	38 (18.1)	60 (17.5)	26 (16.5)	56 (15.3)	
IV	651 (60.5)	113 (53.8)	189 (55.3)	114 (72.2)	235 (64.2)	
ECOG performance status, *N* (%)						0.01
0–1	721 (67.0)	151 (71.9)	241 (70.5)	104 (65.8)	225 (61.5)	
2	324 (30.1)	55 (26.2)	97 (28.4)	45 (28.5)	127 (34.7)	
3	31 (2.9)	4 (1.9)	4 (1.2)	9 (5.7)	14 (3.8)	
Skeletal muscle mass index (kg/m^2^), mean (SD)						<0.001 for all
Overall	8.71 (1.91)	8.92 (1.87)	8.39 (1.96)	9.39 (2.48)	8.31 (1.47)	
Males	9.59 (1.51)	11.67 (0.73)	9.38 (0.87)	12.12 (1.99)	8.89 (1.02)	
Females	7.20 (1.26)	7.95 (0.97)	6.11 (0.51)	7.98 (1.19)	6.11 (0.52)	
Handgrip strength (kg), mean (SD)						<0.001 for all
Overall	25.4 (10.3)	26.9 (8.8)	32.7 (9.9)	17.7 (7.5)	21.1 (7.3)	
Males	30.1 (9.9)	38.2 (6.7)	37.9 (7.0)	25.3 (6.5)	23.1 (6.7)	
Females	18.6 (6.3)	22.8 (5.3)	20.9 (4.3)	13.8 (4.3	13.6 (4.2)	

Abbreviations: ECOG, Eastern Cooperative oncology Group; HG, handgrip strength; IQR, interquartile range; SD, standard deviation; SMMI, skeletal muscle mass index.

^a^ One‐way analysis of variance (continuous variables) or Fisher's exact test (categorical variables).

**FIGURE 1:**
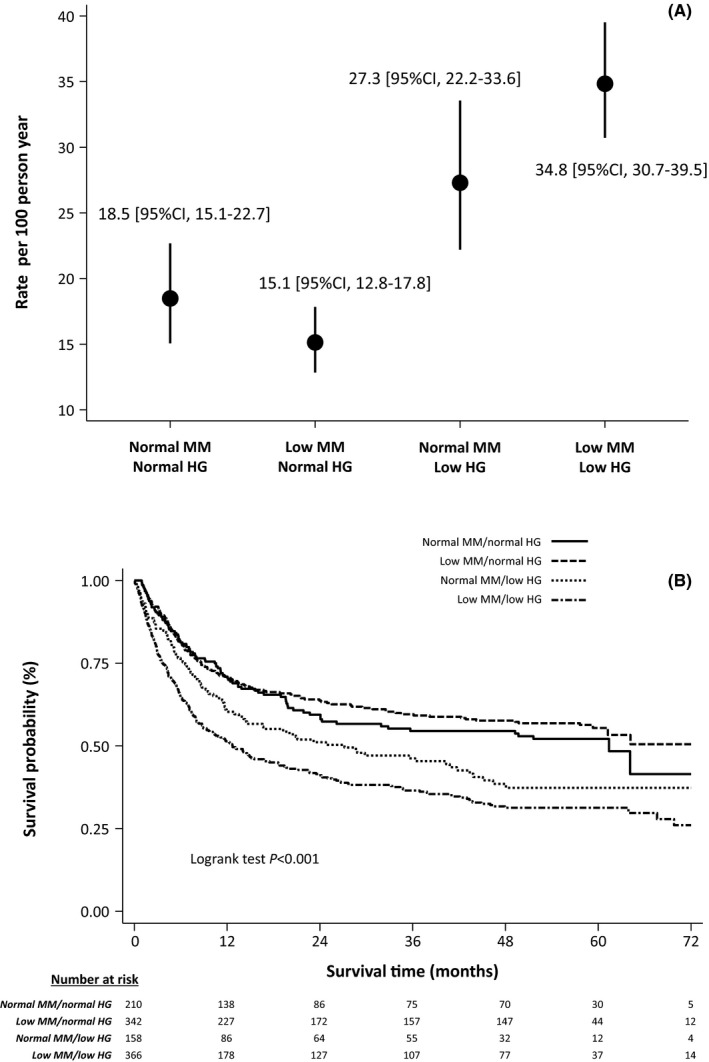
Mortality rates (A) and cumulative survival curves (B) across reduced skeletal muscle mass and muscle weakness strata

**FIGURE 2:**
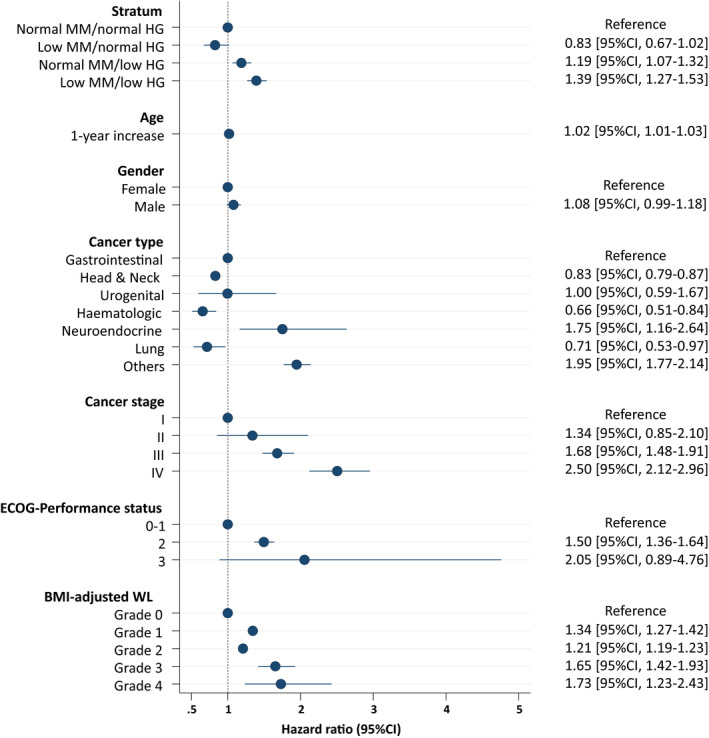
Predictors of mortality (risk estimates by Cox's regression) in the study cohort

After a median follow‐up of 58 months [25th–75th percentile, 37–60], 566 patients had died (mortality rate, 23.0 per 100 person‐year [95% CI, 21.2–25.0]). At univariable analysis both low SMMI and low HG were associated with higher mortality risk: HR = 1.13 [95% CI, 1.04–1.22] (*p* = 0.004) and HR = 1.80 [95% CI, 1.56–2.07] (*p* < 0.001), respectively. A significant qualitative interaction between these two factors was found (*p* < 0.001), with a lower mortality rate in patients with normal HG and low SMMI than in those with normal HG and normal SMMI, and vice versa a higher mortality in patients with low HG and low SMMI than in those with low HG and normal SMMI (Figure 1). This results in an increased risk of death associated with low HG (HR = 1.41, 95% CI 1.28–1.55, *p* < 0.001) in patients with normal SMMI and an even higher risk in patients with low SMMI (HR = 2.02, 95% CI 1.75–2.34, *p* < 0.001). Adjustment for major confounders yielded consistent findings, with an HR associated with a low HG was 1.19 [95% CI 1.07–1.32, *p* = 0.002] in patients normal SMMI and of 1.39 [95% CI 1.27–1.53, *p* = 0.001] in patients with low SMMI (Figure 2).

## 4 DISCUSSION

The present study showed that muscle weakness is a strong independent prognostic factor in patients with cancer, particularly when it coexists with reduced BIA‐estimated SMM (additive effect).

Our results are consistent with recent findings supporting not only the independent negative impact of low muscle strength in different cancer types, but also its additional predictive value of worse outcomes (e.g. perioperative outcome and survival) when combined with reduced lean body mass as estimated by BIA or assessed by CT.^13^, ^14^, ^15^, ^16^, ^17^, ^39^ Burtin et al. found that muscle weakness alone predicted survival in patients with lung cancer and that it significantly worsened prognosis when associated with reduced fat‐free mass, particularly in patients with a good performance status.^14^ Sarcopenia defined by reduced muscle mass (by CT) and strength was also found to predict higher rates of 90‐day morbidity than isolated weakness or low muscle mass after liver resection for malignant tumors.^13^ Muscle weakness has been also proposed as a reliable phenotypic criterion of malnutrition, substitute of reduced muscle mass or arm muscle circumference or fat‐free mass index, showing a stronger prediction of mortality in patients with cancer.^18^, ^19^, ^20^ Indeed, low SMM is a risk factor and not an absolute determinant of worse outcomes (e.g. dose‐limiting toxicity, mortality.) in this patient's population,^40^ even when assessed by CT. We cannot exclude that the additional evaluation of muscle weakness would improve risk assessment and discrimination of patients, thus resulting in a more powerful trigger for guiding tailored and timely interventions consisting of adequate nutritional care and exercise. Further studies in this area are warranted, but it is noteworthy that muscle weakness, with reduced performance, likely denotes refractory cachexia when combined with other nutritional features such as low BMI, WL or reduced muscle mass frequently secondary to variable degree of anorexia and systemic inflammation.^1^, ^2^, ^41^ However, recent studies have shown that upper body strength alone did not discriminate between patients with or without cachexia and those with and without muscle wasting, as the incidence of both may be heterogeneous in different cancer types.^42^, ^43^ On the other hand, although the relationship between muscle mass and muscle function is complex,^44^ it is interesting that muscle dysfunction predicted worse outcome, even in presence of normal muscle mass and independently of multiple confounders. Reduced functional capacity in patients with cancer may be present even in absence of muscle catabolism, likely being an early sign of systemic inflammation, not yet activated within the muscle.^45^, ^46^ Nonetheless, low grip strength has been associated with higher case‐fatality rates in different chronic diseases.^47^ Interestingly, in patients with cancer, anabolic agents have been proven to increase muscle mass but have failed in improving muscle function and performance as well as survival,^44^ while studies addressing the role of exercise interventions––namely prehabilitation, which may include nutrition––have shown not only an increase in strength and performance, but also improved outcome such as lower post‐operative complications, increased treatment‐tolerance, reduced symptoms burden and hospitalisation rates,^48^, ^49^, ^50^, ^51^, ^52^, ^53^ thus supporting the importance of physical functioning. As grip strength is an inexpensive, simple and routinely feasible measurement also in patients with cancer,^21^ it is reasonable to argue for its implementation in the screening and diagnostic approach of sarcopenia in this patient's population. This proposal is consistent with the operational algorithm for case‐finding and confirmation recently implemented for an old age population by expert panels,^12^, ^54^ according to which the evaluation of muscle strength and/or performance should come to the forefront, as some methodologies used in the assessment of body composition––such as BIA and Dual‐Energy X‐ray Absorptiometry––do not directly measure MM.^55^ Indeed, the complexity of sarcopenia in oncology must be acknowledged. To address this issue we have adjusted for multiple confounders, including age, and used age and gender‐specific threshold values. Nowadays, despite the recognised prognostic value of reduced MM in patients with cancer, the evaluation of body composition is still not part of routine care and it is reasonably affected by the availability of technologies and local research interests. Most literature is based on the use of CT, which provides a more accurate assessment of this body compartment and it is not influenced by hydration such as BIA, but it could be performed only at established time points as a standard of care and still lacks of definite threshold values. Furthermore, also age‐specific cut‐offs for SMM assessed by CT are not available. On the other hand, BIA is non‐invasive, inexpensive and, as bedside procedure, can be performed whenever a major event occurs or the conditions of the patient change. In our study we have excluded patients with fluid retention, in order to limit assessment bias, but we recognise the use of BIA as a limitation as more standardisation of its use is needed also to support the inter‐changeability of the different devices. However, our study likely reflects the daily practice as we have analysed data of patients assessed at different moments of their disease's course.

Therefore, a lot of work still needs to be done in this area and confirmatory studies combining the evaluation of muscle weakness with the use of more accurate technologies in the evaluation of MM are clearly warranted. These should consider multiple relevant outcomes, including treatment tolerance. Nonetheless, the evaluation of physical performance and its prognostic value needs to be addressed. In our study measures of this domain (e.g. gait speed) were not available but they could provide a more comprehensive assessment of sarcopenia.

In conclusion, muscle strength was found to be a relevant predictor of survival both in the absence and in the presence of compromised BIA‐estimated SMM and, as an easy measurement to perform, it should be considered as additional key feature of sarcopenia also in patients with cancer. Confirmatory studies are needed to support an improvement in the operational definition of sarcopenia in this patient's population.

## ETHICS STATEMENT

The use of follow‐up data for the present study has been approved by Institutional Ethics Committees. We obtained written informed consent from every patient.

## CONFLICT OF INTEREST

All the authors have no relationship to disclose.

## Data Availability

Data described in the manuscript, code book and analytic code will not be made available because a specific note was not included in the informed consent at the time of protocol approval and recruitment. Therefore, data sharing was not approved by the Ethics Committees.
